# Association between small dense LDL levels and hepatic fibrosis in patients with nonalcoholic fatty liver disease

**DOI:** 10.1097/MD.0000000000030527

**Published:** 2022-09-16

**Authors:** Sun Young Kim, Subin Mun, Jung Hwan Yu, Young-Joo Jin, Young Ju Suh, Sang-Heon Cho, Jin-Woo Lee

**Affiliations:** a Department of Internal Medicine, Inha University Hospital, Inha University School of Medicine, Incheon, Korea; b Digestive Disease Center, Department of Internal Medicine, Inha University School of Medicine, Incheon, Korea; c Department of Biomedical Sciences, College of Medicine, Inha University, Incheon, Korea; d Department of Clinical Pharmacology, Inha University Hospital, Inha University School of Medicine, Incheon, Korea.

**Keywords:** low density lipoproteins, nonalcoholic fatty liver disease, steatohepatitis

## Abstract

While patients with nonalcoholic fatty liver disease (NAFLD) continue to increase worldwide, few hematological biomarkers are helpful. This study examined the potential of small dense low density lipoprotein (sdLDL) as a noninvasive biomarker for NAFLD and investigated the relevance of liver fibrosis. One hundred seventy two patients were enrolled: 121 NAFLD patients and 51 healthy controls. The lipoprotein profiles of NAFLD patients and controls were analyzed, and transient elastography (Fibroscan®) was performed to evaluate the degree of NAFLD. The liver biopsy results in some NAFLD patients were also analyzed. Age-gender matching was performed among the 172 patients, and a comparison with 46 NAFLD patients with the control group confirmed that the sdLDL (*P* < .001) is significantly higher in the NAFLD group. A liver fibrosis test performed on 121 NAFLD patients confirmed a positive correlation between the degree of hepatic fibrosis and the sdLDL/LDL ratio (*R* = 0.215, *P = *.017). The area under the curve of the sdLDL for the diagnosis of NAFLD was 0.734 (95% CI, 0.631–0.838), and the area under the curve of the sdLDL/LDL ratio was 0.730 (95% CI, 0.621–0.829). The sdLDL and NAFLD activity scores of the 11 NAFLD patients who underwent liver biopsy showed a positive correlation, but it was not statistically significant. The sdLDL was higher in NAFLD patients than in controls and showed a tendency to increase gradually with increasing degree of hepatic steatosis and fibrosis. In particular, the sdLDL/LDL ratio showed a significant correlation with the degree of hepatic fibrosis, and the sdLDL measurement could be useful in NAFLD patients.

## 1. Introduction

Along with obesity, the prevalence of nonalcoholic fatty liver disease (NAFLD) is increasing worldwide and currently accounts for about 25% of the world’s population.^[1]^ Therefore, the burden of the healthcare system for the management and treatment of NAFLD is increasing and becoming a serious healthcare issue. Patients with NAFLD have more frequent metabolic comorbidities, such as obesity, diabetes, and dyslipidemia, and the comorbidity of these metabolic diseases is closely related to the prognosis of NAFLD patients.^[2,3]^ Therefore, it is necessary to recognize NAFLD as part of metabolic syndrome rather than as an independent disease, and investigating the association between NAFLD and associated metabolic syndrome can also help broaden the perspective on NAFLD and understand NAFLD.

Dyslipidemia is one of the most common comorbidities in patients with NAFLD and is considered the cause of the increase in cardiovascular complications in patients with NAFLD.^[4]^ Thus far, several studies have confirmed an increase in small dense low density lipoprotein (sdLDL) particles, a subtype of low density lipoprotein (LDL), in patients with NAFLD, and an increase of the sdLDL levels has been observed in patients with advanced fibrosis compared to patients with simple steatosis.^[5–7]^ On the other hand, the mechanism by which sdLDL increases in NAFLD patients and how sdLDL differs from that of normal individuals is unclear. A recently reported study showed a positive correlation between the degree of hepatic fibrosis and sdLDL in NAFLD patients, but the degree of hepatic fibrosis was based on a liver fibroscan test (Fibroscan®) rather than a liver biopsy.^[8]^ Therefore, more studies will be needed to elucidate the relationship between sdLDL and NAFLD patients and to determine if sdLDL may be useful in NAFLD patients.

Thus far, studies on sdLDL have focused mainly on the risk of cardiovascular disease (CVD) related to atherosclerosis. On the other hand, a study of sdLDL could help diagnose NAFLD or evaluate the degree of fibrosis indirectly in NAFLD patients, given that NAFLD is also associated with metabolic syndrome and is accompanied mainly by dyslipidemia. Therefore, this study examined the change in sdLDL in NAFLD patients by comparing it to normal individuals to understand the relationship between sdLDL and NAFLD using liver biopsy results, albeit in some patients. Finally, the possibility of using sdLDL as a noninvasive biomarker that can be used clinically in NAFLD patients was evaluated.

## 2. Materials and Methods

### 2.1. Study subjects

Patients recruited for this study were recruited from outpatients diagnosed with NAFLD who visited the Department of Gastroenterology, Inha University Hospital, from January 2018 to February 2021. The inclusion criteria were adults aged 18 years or older diagnosed with NAFLD and voluntarily agreed to participate in the study through written consent. NAFLD is a fatty liver disease that is not caused by alcohol intake (20 g/day or less for women, 30 g/day or less for men). The disease was defined from imaging tests in this study. The exclusion criteria included patients with liver diseases other than NAFLD (viral hepatitis, toxic hepatitis, and autoimmune hepatitis), malignancies including hepatocellular carcinoma (HCC), and underlying diseases that may affect the evaluation of fatty liver (e.g., CVD, chronic kidney disease, and chronic lung disease), and immune diseases were excluded. In addition, patients who received treatment that could affect the liver function tests within 1 month before the study and patients who took drugs that could cause fatty liver disease within 3 months before the study were excluded. Patients who had not received transient elastography (TE), had not undergone lipoprotein profile analysis, or had not provided written consent were also excluded. One hundred twenty-one patients with NAFLD were finally included. One hundred seventy-two patients, including 51 healthy male controls who underwent medical examinations at Inha University Hospital, were enrolled in the study.

The basic patient information used in the study (the patient’s body mass index, alcohol history, and other medical history) was confirmed through medical interviews and physical examinations. Biochemical tests, basic blood tests, and lipoprotein profile tests were performed. This study was approved by the Inha University Hospital Institutional Review Board (approval number: INHAUH 2021-07-018-000).

### 2.2. Liver biopsy

Among the 121 NAFLD patients, 11 with informed consent underwent liver biopsies. The minimal essential findings for a diagnosis of nonalcoholic steatohepatitis (NASH) included steatosis, balloon degeneration of hepatocytes, and inflammation in the hepatic lobules. The NASH Clinical Research Network devised the nonalcoholic fatty liver disease activity score (NAS) in 2005 to classify the NAFLD grades. NAS ≥ 5 showed that it is almost consistent with the diagnosis of NASH, but if NAS < 3, it is mostly not NASH, and a score of 3–4 is borderline. On the other hand, NAS is meaningful in measuring the changes in the pathological findings as a response to treatment rather than the pathological diagnosis of NASH itself. It can be measured easily with commonly used H–E staining and Masson trichrome staining. Therefore, it is currently widely used. In the present study, liver samples were obtained using 18 G disposable needles, fixed with 4% formalin, and embedded in paraffin. The degree of fibrosis was classified according to Masson trichrome staining, and the degree of fibrosis was evaluated using a 5-point scale proposed by Brunt and modified by Kleiner et al.

### 2.3. Analysis of lipoprotein profile

Twelve distinct lipoprotein subclasses were evaluated, including very-low-density lipoproteins, 3 intermediate-density lipoproteins (IDL-A, IDL-B, and IDL-C), 7 low-density lipoproteins (LDL-1, LDL-2, LDL-3, LDL-4, LDL-5, LDL-6, LDL-7), and high density lipoprotein (HDL). LDL can be classified into 7 subfractions, from LDL-1 to LDL-7. LDL subclasses 3–7 correspond to small dense LDL subfractions. As in a previous study, the LDL subfractions were analyzed using 3% polyacrylamide gel tube electrophoresis (Lipoprint™ LDL System; Quantimetrix, Redondo Beach, CA). The electrophoretic mobility (Rf) was also calculated using the lipiprint LDL system template and Lipoware software (property of Quantimetrix, Redondo Beach, CA). The Rf of the LDL subfraction was estimated using the Rf between the ultra-low-density lipoprotein fraction (Rf 0.0) and HDL fraction (Rf 1.0). The 7 low-density lipoproteins were classified as Rfs = 0.32, 0.38, 0.45, 0.51, 0.56, 0.60, and 0.64, respectively. The lipoproteins were classified as Rfs = 0.32, 0.38, 0.45, 0.51, 0.56, 0.60, and 0.64, respectively. Blood tests were performed on 51 healthy control volunteers to investigate the normal range of sdLDL levels using immunoturbidity analysis. The expected normal value, which is defined as the 95% CI (mean ± 2SD) for the sdLDL levels, was calculated as 0 to 6.3 mg/dL.

### 2.4. Noninvasive tests for the diagnosis of steatosis

NAFLD severity was evaluated as a widely used indicator for diagnosing hepatic steatosis. The NAFLD severity was assessed by noninvasive methods, such as TE (Fibroscan®), liver stiffness (LS), and controlled attenuation parameter (CAP) scores measured by TE and fatty liver index. Based on the serological examination, steatosis was diagnosed, and the degree of steatosis was predicted. According to age, sex, body mass index (BMI), and biochemical tests, steatosis could be excluded if the hepatic steatosis index was less than 30. On the other hand, steatosis could be diagnosed with a high predictive value if it was over 36.

Noninvasive methods for diagnosing liver fibrosis include liver fibrosis scans, NAFLD fibrosis score (NFS), FIB-4 index, and APRI (AST to Platelet Ratio Index). NFS consists of age, fasting blood glucose, BMI, platelet, albumin, and aspartate aminotransferase (AST)/alanine aminotransferase (ALT). FIB-4 consists of age, platelet, AST, and ALT. Therefore, it can be calculated relatively simply. The correlation between the sdLDL level measured through blood tests and the LS, CAP, and APRI was evaluated. The correlation between the sdLDL level and the NAFLD severity was investigated.

### 2.5. Statistical analysis

This study performed statistical analysis using SPSS software (version 26, Armonk, NY). Pearson correlation and Spearman correlation were used as parametric and nonparametric methods to confirm the correlation between sdLDL and hepatic stevis and fibrosis. Continuous variable analysis was performed using a *t* test and Mann–Whitney test, and the categorical variables were analyzed using Pearson’s chi-square test. A *P*-value < .05 was considered significant.

## 3. Results

### 3.1. Baseline characteristics

From January 2018 to February 2021, 121 patients with NAFLD were enrolled, and 51 healthy men who underwent medical examinations at Inha University Hospital were registered as controls. Finally, 172 people were included in the study. Table 1 lists the clinical and laboratory characteristics of the patients. Approximately 23% of NAFLD patients had diabetes mellitus, and 22% had hypertension. The average BMI of the patients was 30.81, mostly overweight or obese, and controls group was 24.49. In the basic blood tests, the white blood cell was slightly higher in the NAFLD patients than controls (7020 vs 6454/µL, *P* = .042), but platelets were not different (255,733 vs 272,137/µL, *P* = .105). In the biochemical tests, total bilirubin (0.69 vs 0.59/µL, *P* = .042), AST (51 vs 18 IU/L, *P* < .001), ALT (76 vs 21 IU/L, *P* < .001), total cholesterol (199 vs 174 mg/dL *P* < .001), LDL (116 vs 96 mg/dL, *P* < .001), and sdLDL levels (12.20 vs 4.25 mg/dL, *P *< .001) were significantly higher in the NAFLD group. There was no difference in the HDL (42 vs 45 mg/dL, *P* = .071) between the 2 groups. The detailed lipid profile values and differences between the 2 groups are presented in the Table S1, Supplemental Digital Content 1, http://links.lww.com/MD/H284.

**Table 1:** Clinical and laboratory characteristics of the subjects.

Characteristics	Total (n = 172)	NAFLD (n = 121)	Control (n = 51)	*P* value
Age (yr)	39.25 ± 12.51	42.03 ± 13.36	32.65 ± 6.6	<.001
Male, n (%)	126 (73.25%)	75 (61.98%)	51 (100%)	<.001
Hypertension, n (%)	27 (15.69%)	27 (22.31%)	0 (0%)	
DM	28 (16.27%)	28 (23.14%)	0 (0%)	
BMI (kg/m^2^)	28.87 ± 7.49	30.81 ± 8.07	24.49 ± 2.86	<.001
LS (kPa)	6.1 ± 2.66	6.1 ± 2.66		
CAP (dB/m)	318.3 ± 39.43	318.3 ± 39.43		
WBC (/µL)	6851 ± 1664	7020 ± 1726	6454 ± 1448	.042
Platelet (/µL)	260626 ± 60417	255733 ± 59845	272137 ± 60781	.105
Total bilirubin (mg/dL)	0.66 ± 0.31	0.69 ± 0.32	0.59 ± 0.28	.042
AST (IU/L)	41 ± 38.99	51 ± 42.57	18 ± 8.57	<.001
ALT (IU/L)	60 ± 61.63	76 ± 66.38	21 ± 14.92	<.001
Total cholesterol (mg/dL)	190 ± 39.25	197 ± 40.19	174 ± 31.70	<.001
LDL (mg/dL)	110 ± 29.57	116 ± 29.55	96 ± 24.80	<.001
HDL (mg/dL)	43 ± 9.70	42 ± 9.82	45 ± 9.17	.071
sdLDL (mg/dL)	9.85 ± 12.12	12.20 ± 13.01	4.25 ± 7.17	<.001

Data are mean ± SD.

ALT = alanine aminotransferase, AST = aspartate aminotransferase, BMI = body mass index, CAP = controlled attenuation parameter, DM = diabetes mellitus, HDL = high density lipoprotein, LDL = low density lipoprotein, LS = liver stiffness, SD = standard deviation, sdLDL = small dense low density lipoprotein, WBC = white blood cell.

### 3.2. Comparison of sdLDL between normal controls and NAFLD patients after age-sex match

**Figure 1.**
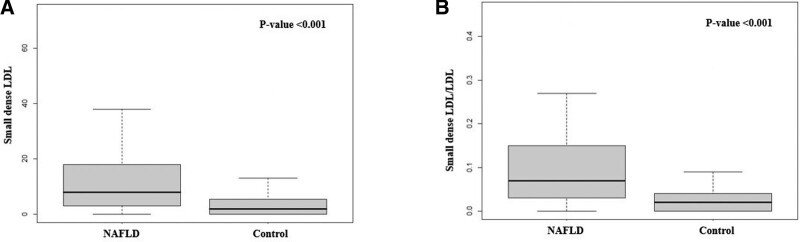
Comparison of sdLDL between normal controls and NAFLD patients. (A) Comparison of sdLDL between normal controls and NAFLD patients. (B) Comparison of sdLDL/LDL ratio between normal controls and NAFLD patients. HDL = high density lipoprotein, LDL = low density lipoprotein, NAFLD = nonalcoholic fatty liver disease, sdLDL = small dense low density lipoprotein.

A significant difference in age and sex was noted in all patient groups, so match analysis was re-executed by correcting for age and sex (Table 2). As a result, 19% (n = 9) of NAFLD patients had diabetes mellitus, and 19% (n = 9) had hypertension; the average BMI of the NAFLD patients was 33.1 (vs control group 24.6). In the biochemical tests, AST (54 v*s* 19 IU/L, *P* < .001), ALT (93 v*s* 20 IU/L, *P* < .001), LDL (120 vs 97 mg/dL, *P* < .001), and sdLDL levels (14.9 vs 4.6 mg/dL, *P *< .001) were significantly higher in the NAFLD group. The sdLDL/LDL ratio (0.10 vs 0.04, *P* < .001) also were significantly higher in the NAFLD group than the age-sex match control group (Fig. 1).

**Table 2:** Clinical and laboratory characteristics of the age-sex matched.

Characteristics	Total (n = 92)	NAFLD (n = 46)	Control (n = 46)	*P* value
Age (yr)	33.44 ± 6.47	33.46 ± 6.45	33.43 ± 6.47	.987
Male, n (%)	100%	46 (100%)	46 (100%)	
Hypertension, n (%)	9 (9.78%)	9 (19%)	0 (0%)	<.001
DM	9 (9.78 %)	9 (19%)	0 (0%)	<.001
BMI (kg/m^2^)	28.76 ± 9.56	33.1 ± 11.86	24.63 ± 2.8	<.001
LS (kPa)		6.1 ± 2.55		
CAP (dB/m)		327.2 ± 40.3		
WBC (/µL)	6854 ± 1620	7256 ± 1679	6460 ± 1452	.018
Platelet (/µL)	259725 ± 57752	249711 ± 56737	269521 ± 57037	.102
Total bilirubin (mg/dL)	0.68 ± 0.32	0.8 ± 0.33	0.59 ± 0.29	.004
AST (IU/L)	36 ± 33.79	54 ± 39.45	18 ± 8.21	<.001
ALT (IU/L)	56 ± 67.28	92 ± 79.04	20 ± 13.99	<.001
Total cholesterol (mg/dL)	187 ± 37.24	200 ± 37.55	174 ± 32.7	.03
LDL (mg/dL)	108 ± 29.08	120 ± 27.41	97 ± 25.85	<.001
HDL (mg/dL)	42 ± 8.92	40 ± 8.65	44 ± 8.82	.029
sdLDL (mg/dL)	9.72 ± 13.21	14.9 ± 15.43	4.6 ± 7.47	<.001

Data are mean ± SD.

ALT = alanine aminotransferase, AST = aspartate aminotransferase, BMI = body mass index, CAP = controlled attenuation parameter, DM = diabetes mellitus, HDL = high density lipoprotein, LDL = low density lipoprotein, LS = liver stiffness, NAFLD = nonalcoholic fatty liver disease, SD = standard deviation, sdLDL = small dense low density lipoprotein, WBC = white blood cell.

### 3.3. Correlation between sdLDL level and NAFLD severity

**Figure 2.**
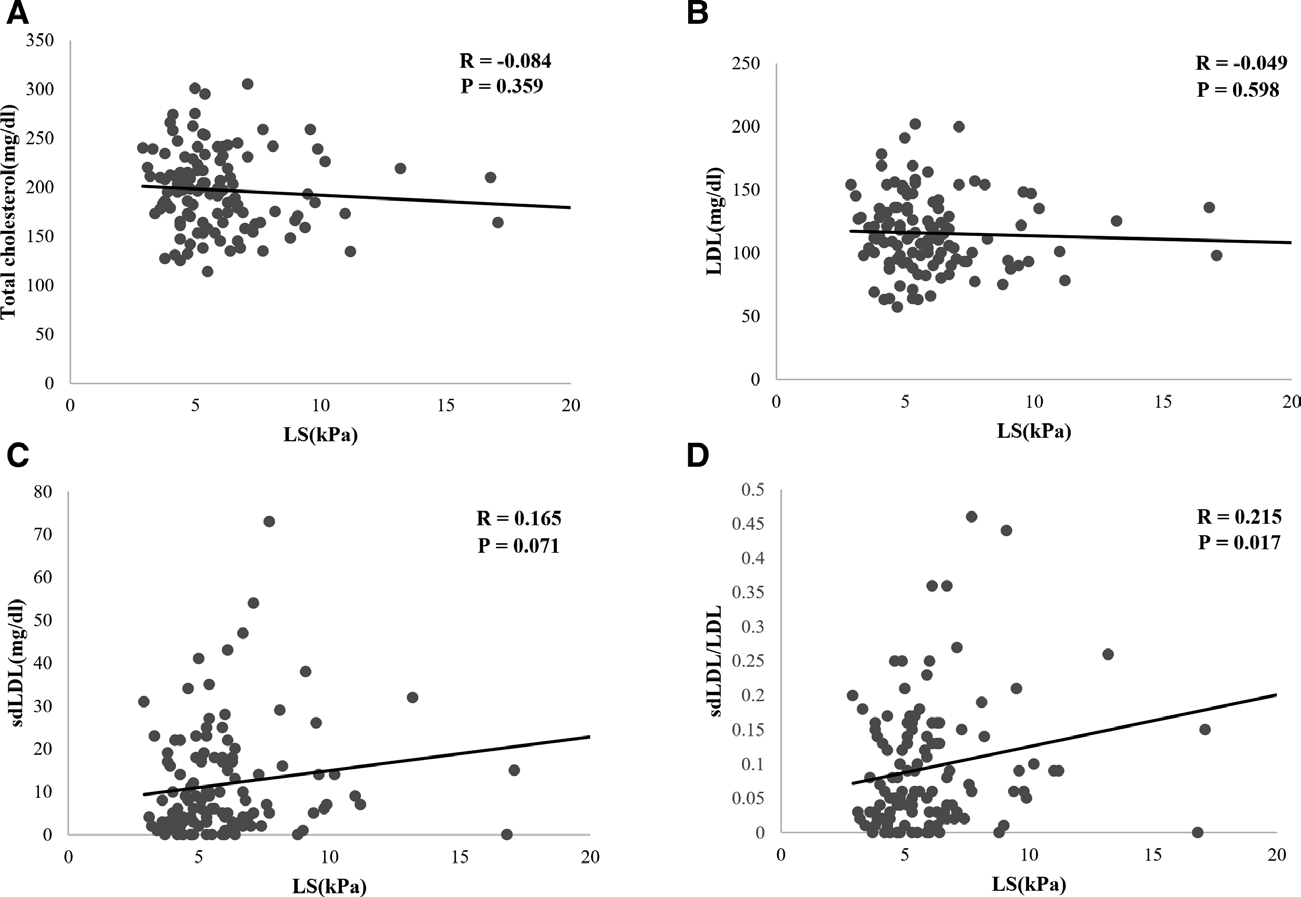
Correlation between lipid profile and LS. (A) Correlation between total cholesterol and LS. (B) Correlation between total LDL and LS. (C) Correlation between sdLDL and LS. (D) Correlation between sdLDL/LDL ratio and LS. HDL = high density lipoprotein, LDL = low density lipoprotein, LS = liver stiffness, sdLDL = small dense low density lipoprotein.

TE (Fibroscan®) was performed to assess the NAFLD severity. In the present study, the LS cut-off values for fibrosis F2, fibrosis F3, and fibrosis F4 in NAFLD patients were 7, 8.7, and 10.3, respectively, depending on the reference.^[8]^ The correlation between NAFLD severity and lipoprotein profile was analyzed. The correlation between the lipoprotein profile and NAFLD severity was analyzed using LS obtained by TE (Fibroscan®). The total cholesterol and LDL levels showed a negative correlation with LS, but were not significant (R = –0.084, *P* = .359 for the total cholesterol; R = –0.049, *P *= .598 for LDL). sdLDL showed a positive correlation with LS, but the correlation was not significant, and sdLDL/LDL showed a significant positive correlation with LS (*R* = 0.2158, *P* = .0178) (Fig. 2). The correlation between the lipoprotein profile and NAFLD severity was analyzed using CAP obtained from TE (Fibroscan®). The total cholesterol level had a negative correlation with CAP, but they were not significant. sdLDL and sdLDL/LDL had a positive correlation with CAP, but they were not statistically significant (Figure S1, Supplemental Digital Content 2, http://links.lww.com/MD/H285).

**Figure 3.**
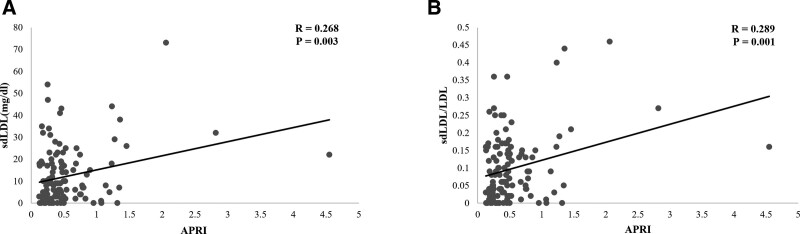
Correlation between lipid profile and APRI. (A) Correlation between sdLDL and APRI. (B) Correlation between sdLDL/LDL ratio and APRI. APRI = AST to platelet ratio index, LDL = low density lipoprotein, sdLDL = small dense low density lipoprotein.

Studies have shown that APRI is an easy-to-use score as a reliable predictor of CVD in metabolic patients.^[9]^ Therefore, this study investigated the association between sdLDL (or sdLDL ratio) and APRI. A statistically significant positive correlation was observed between APRI and sdLDL (*R* = 0.268, *P *= .003). Furthermore, a statistically significant positive correlation was noted between APRI and sdLDL/LDL ratio (*R* = 0.289, *P *= .001) (Fig. 3).

### 3.4. Correlation between sdLDL level and NAFLD activity score (NAS)

**Figure 4.**
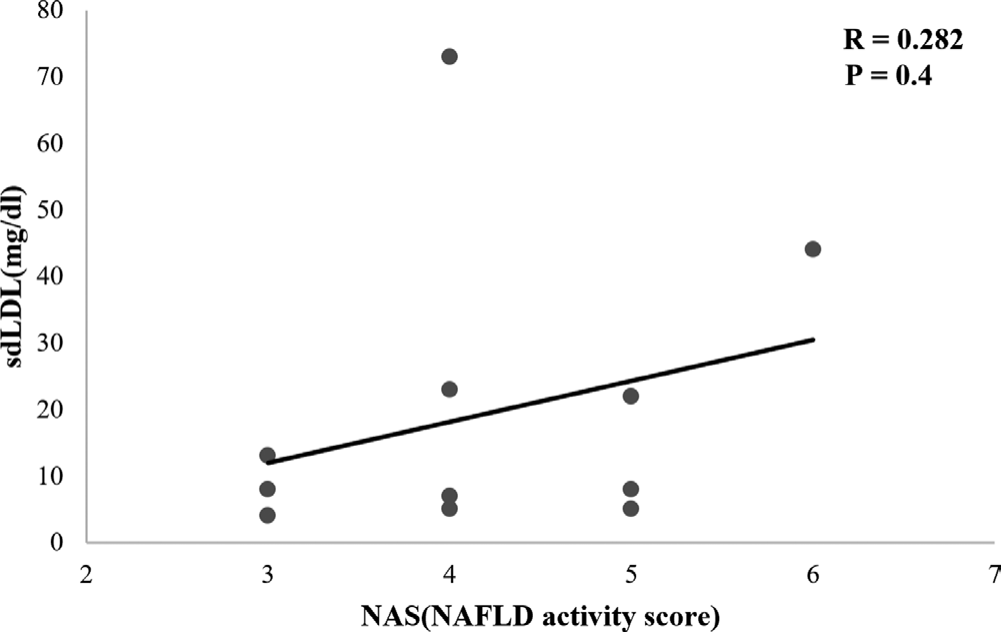
Correlation between sdLDL level and NAFLD activity score (NAS). NAFLD = nonalcoholic fatty liver disease, sdLDL = small dense low density lipoprotein.

The NAS scores of NAFLD patients who underwent biopsy were analyzed to compare liver biopsy results with sdLDL results. The biopsies of 11 patients were confirmed, and the average NAS score was 4.2. Looking at each, 3 people had NAS 3 values, 4 people had NAS 4 values, 3 people had NAS 5 values, and 1 person had NAS 6 values. In this study, the sdLDL and NAFLD activity scores (NAS) of the NAFLD patients showed a positive correlation, but the correlation was not statistically significant, possibly because of the small number of patients who underwent a biopsy (Fig. 4).

### 3.5. Possibility of sdLDL for NAFLD diagnosis

**Figure 5.**
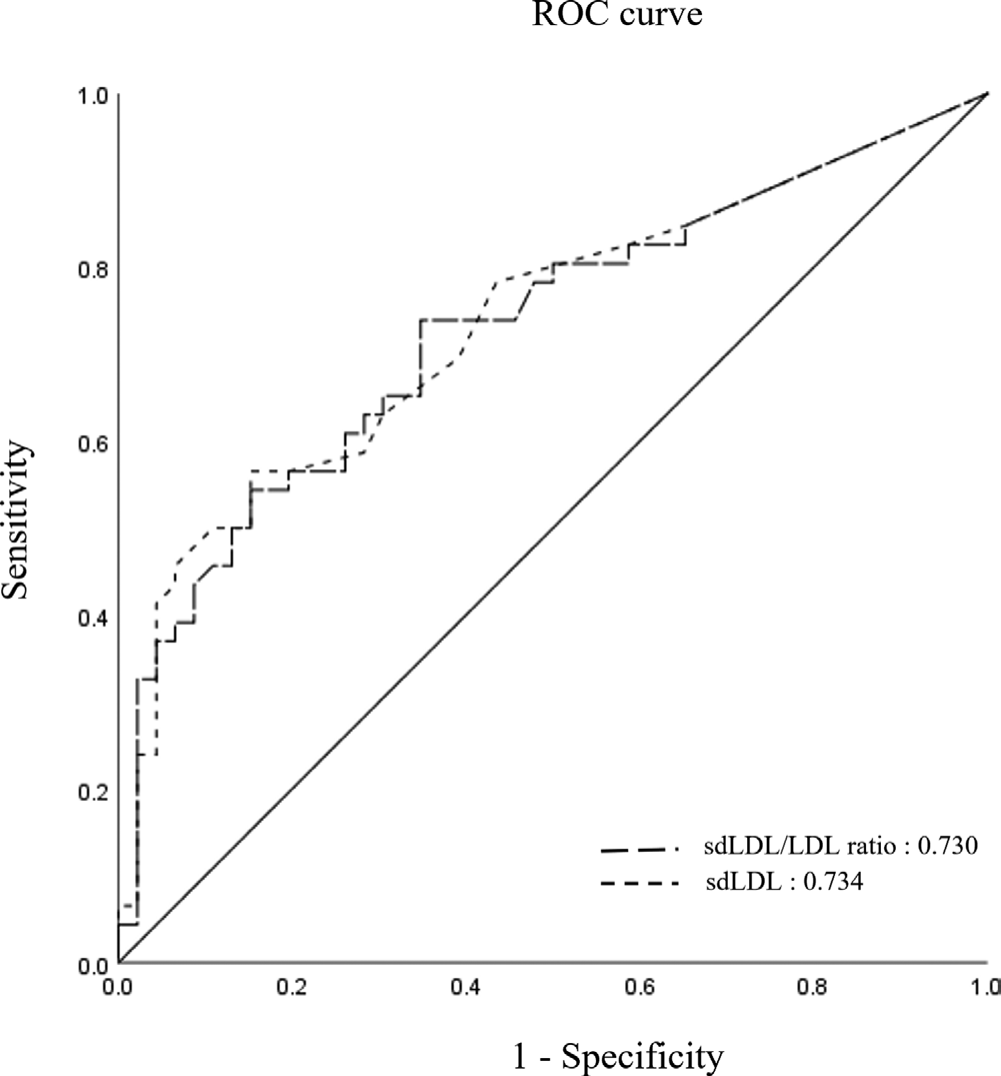
The AUC value of sdLDL level for the diagnosis of NAFLD. The AUC value of sdLDL level for the diagnosis of NAFLD was 0.734 (95% CI 0.631–0.838), and the AUC value of the sdLDL/LDL ratio was 0.730 (95% CI 0.621–0.829). AUC = area under the curve, LDL = low density lipoprotein, NAFLD = nonalcoholic fatty liver disease, sdLDL = small dense low density lipoprotein.

The applicability of sdLDL for NAFLD diagnosis was evaluated based on the sdLDL results of the male control and NAFLD patients. When the sdLDL cut-off value for the diagnosis of NAFLD was set to 2.5 mg/dL, the sensitivity and specificity corresponded to 78% and 57%, respectively. On the other hand, when the sdLDL cut-off value was 9 mg/dL or higher, the sensitivity was 56.5%, and the specificity was 84.8%. Furthermore, the area under the curve of the sdLDL level for the diagnosis of NAFLD was 0.734 (95% CI 0.631–0.838), and the area under the curve value of the sdLDL/LDL ratio was 0.730 (95% CI 0.621–0.829) (Fig. 5). Although the ability of sdLDL to discriminate against NAFLD in this study is not excellent, it is believed to be an acceptable value, and it is expected that it can be used as a better NAFLD diagnostic marker if combined with several NAFLD biomarkers in future studies.

## 4. Discussion

In our study, sdLDL and sdLDL/LDL ratio were significantly higher in NAFLD patients than in healthy controls. Additionally, sdLDL and sdLDL/LDL ratios showed a tendency to increase gradually with an increasing degree of hepatic steatosis and fibrosis based on TE (Fibroscan®). In particular, the sdLDL/LDL ratio showed a significant correlation with the degree of liver fibrosis. A previous study has also reported a positive correlation between sdLDL and LS levels, which indicate the degree of hepatic fibrosis, in patients with NAFLD.^[8]^ In addition, the sdLDL/LDL ratio showed a positive correlation with fatty liver index, a diagnostic indicator of hepatic steatosis. However, the previous study had a disadvantage in that the number of subjects was small and that a liver biopsy was not performed to evaluate liver fibrosis. In addition, there was no control group in the previous study, making it impossible to confirm the difference in sdLDL between healthy control and NAFLD patients. Therefore, in the present study, we tried to overcome these limitations of these.

NAFLD is a progressive disease from NAFL to cirrhosis. It can progress to NASH and fibrosis, ultimately leading to cirrhosis and hepatocellular carcinoma.^[9]^ Liver fibrosis is the most important factor in determining long-term prognosis, such as the occurrence of HCC and liver-related death. It is becoming increasingly important to identify the improvement and progression of hepatic fibrosis through serial assessments as well as treatment.^[10]^ Methods of evaluating liver fibrosis in nonalcoholic fatty liver diseases include imaging, serological tests, and liver biopsy.^[11]^ Because it is not easy to perform liver biopsy for all patients in actual clinical practice, the high-risk group with advanced liver fibrosis can be differentiated by confirming the preferentially performed tests, such as TE (Fibroscan®), FIB-4, and NFS test.^[10]^ In addition, the methods for measuring liver fibrosis, such as point shear wave elastography,^[12]^ 2D shear wave elastography,^[13]^ magnetic resonance elastography (MRE) have also been used recently. MRE has high diagnostic ability for diagnosing hepatic fibrosis, so the degree of hepatic fibrosis can be measured throughout the liver.^[14]^ However, MRE also has a disadvantage in that it is difficult to use it universally in all medical institutions due to accessibility problems such as a high cost.

In this study, TE (Fibroscan®) was used as a test method to evaluate hepatic steatosis and fibrosis. TE (Fibroscan®) shows high sensitivity and specificity for diagnosing liver fibrosis in patients with NAFLD through quick and simple noninvasive measurements of liver elasticity.^[15]^ In addition, noninvasive fibrosis scores using clinical parameters and serological indicators, such as APRI and FIB-4, were used to assess the severity of NAFLD.^[11]^ Liver biopsies were performed to determine the stage of steatohepatitis and fibrosis with the consent of some patients with NAFLD. Because the purpose of this study was to evaluate the potential of sdLDL as a noninvasive biomarker for NAFLD, this study variously examined the relationship between sdLDL and fibrosis grade. By comparing lipoprotein profiles of healthy controls and NAFLD patients, it was confirmed that sdLDL levels were significantly different between control and NAFLD patients. In addition, it was confirmed that there was a significant correlation between the degree of liver fibrosis and the sdLDL/LDL ratio in NAFLD patients.

sdLDL is a subclassification of LDL cholesterol related to metabolic diseases.^[16]^ Many studies on sdLDL have focused on the risk of CVD and the incidence of atherosclerosis.^[17]^ Some studies have shown that sdLDL levels are associated with the severity of CVD.^[18]^ In NAFLD patients, CVD is one major comorbidity and a major cause of death associated with NAFLD. Therefore, the degree of sdLDL might be related to the severity of CVD in NAFLD patients.^[18]^ The risk of CVD is increased when the NFS or liver biopsy is accompanied by advanced liver fibrosis.^[19–21]^ In addition to NAFLD severity, sdLDL levels have an important influence on the complications of atherosclerotic diseases including CVD in NAFLD patients. sdLDL may be a noninvasive biomarker in NAFLD patients because the sdLDL/LDL ratio showed a significant correlation with liver fibrosis. Therefore, a follow-up study is needed to observe the correlation between sdLDL levels in NAFLD patients and CVD comorbidities.

This study has some limitations. First, it was a single-center study which might not reflect the global situation. In addition, this study had a small number of controls with all subjects being males in the control groups. Therefore, in order to establish the results of this study more clearly, it is necessary to conduct multicenter studies including more samples and validation in various countries. Gender differences could not be completely excluded when comparing basic clinical characteristics of the control group and NAFLD patients. Therefore, this study tried to overcome this limitation by performing age-gender matching analysis. Second, as there were few subjects who underwent biopsy, the sdLDL level showed a positive correlation with steatohepatitis or fibrosis, but there was no statistical significance. Of course, more biopsy results are needed to draw clear conclusions, but in actual clinical practice, liver biopsy in NAFLD patients is limited for several reasons, including patient compliance and complication such as bleeding. Therefore, in this study, to understand the relationship between sdLDL and liver fibrosis, we tried to determine the degree of fibrosis using various methods such as liver fibrosis test (Fibroscan®) and fibrosis predictive panels (APRI and FIB-4).

## 5. Conclusion

In conclusion, the sdLDL and sdLDL/LDL ratios were significantly higher in NAFLD patients than the normal controls and showed a tendency to increase gradually according to the degree of hepatic steatosis and fibrosis. In particular, the sdLDL/LDL ratio showed a significant correlation with the degree of hepatic fibrosis, suggesting its potential use as a serological marker for predicting advanced liver fibrosis and diagnosing NAFLD.

## Author contributions

**Conceptualization:** Jung Hwan Yu.

**Data curation:** Sun Young Kim, Subin Mun, Jung Hwan Yu.

**Formal analysis:** Young Ju Suh.

**Funding acquisition:** Jung Hwan Yu.

**Investigation:** Sun Young Kim, Jung Hwan Yu.

**Methodology:** Sun Young Kim, Jung Hwan Yu.

**Project administration:** Sun Young Kim, Jung Hwan Yu.

**Resources:** Sun Young Kim, Jung Hwan Yu.

**Software:** Sun Young Kim, Jung Hwan Yu.

**Supervision:** Jung Hwan Yu.

**Validation:** Young Ju Suh.

**Writing – original draft:** Sun Young Kim.

**Writing – review & editing:** Jung Hwan Yu.

## Supplementary Material


