# Supplementary material for: Association between small dense LDL levels and hepatic fibrosis in patients with nonalcoholic fatty liver disease
Source: Medicine (Baltimore). 2022 Sep 16;101(37):e30527. doi: 10.1097/MD.0000000000030527 (PMC9478249; doi:10.1097/MD.0000000000030527)
Supplement: Supplementary file 1 [file medi-101-e30527-s001.pdf]

Supplement Table 1. Lipid profile of the subjects

Lipid	Total(n=172)	NAFLD(n=121)	Control(n=51)	Value
Total cholesterol (mg/dL)	190.03±39.25	196.96±40.19	173.68±31.70	<0.001
TG (mg/dL)	170.41±77.08	170.41 ±77.08		
LDL (mg/dL)	109.95±29.58	115.81±29.56	96.05±24.80	<0.001
VLDL (mg/dL)	36.98±11.59	38.89±11.46	32.43±10.71	<0.001
IDL-A (mg/dL)	13.29±4.55	13.21±4.37	13.47±4.99	0.738
IDL-B (mg/dL)	9.2±3.63	9.61±3.77	8.22±3.11	0.021
IDL-C (mg/dL)	20.09±5.63	20.45±6.05	19.25±4.43	0.153
LDL-1 (mg/dL)	32.29±10.52	32.29±10.86	32.29±9.77	0.998
LDL-2 (mg/dL)	25.17±11.29	27.96±10.2	18.55±11.10	<0.001
LDL-3 (mg/dL)	7.54±7.49	9.21±7.83	3.57±4.73	<0.001
LDL-4 (mg/dL)	1.68±3.63	2.21±4.05	0.41±1.80	<0.001
LDL-5 (mg/dL)	0.46±2.60	0.57±3.01	0.2±1.15	0.240
LDL-6 (mg/dL)	0.17±1.5	0.21±1.75	0.08±0.56	0.471
LDL-7 (mg/dL)	0±0	0±0	0±0	
sdLDL (mg/dL)	9.84±12.12	12.21±13.01	4.25±7.16	<0.001
sdLDL/LDL ratio	0.08±0.09	0.098±0.096	0.04±.0.06	<0.001

Data is mean±SD.

HDL=high density lipoprotein, IDL=intermediate density lipoprotein, LDL=low density lipoprotein, sdLDL=small density low density lipoprotein, TG=triglyceride, VLDL=very low

density lipoprotein.
