# Supplementary material for: Association between small dense LDL levels and hepatic fibrosis in patients with nonalcoholic fatty liver disease
Source: Medicine (Baltimore). 2022 Sep 16;101(37):e30527. doi: 10.1097/MD.0000000000030527 (PMC9478249; doi:10.1097/MD.0000000000030527)

Supplementary Fig 1. Correlation between lipid profile and CAP

(A) Correlation between total cholesterol and CAP. (B) Correlation between total LDL and CAP. (C) Correlation between sdLDL and CAP. (D) Correlation between sdLDL/LDL ratio and CAP.

CAP, Controlled attenuation parameter; LDL, low density lipoprotein; HDL, high density lipoprotein; sdLDL, small dense low density lipoprotein

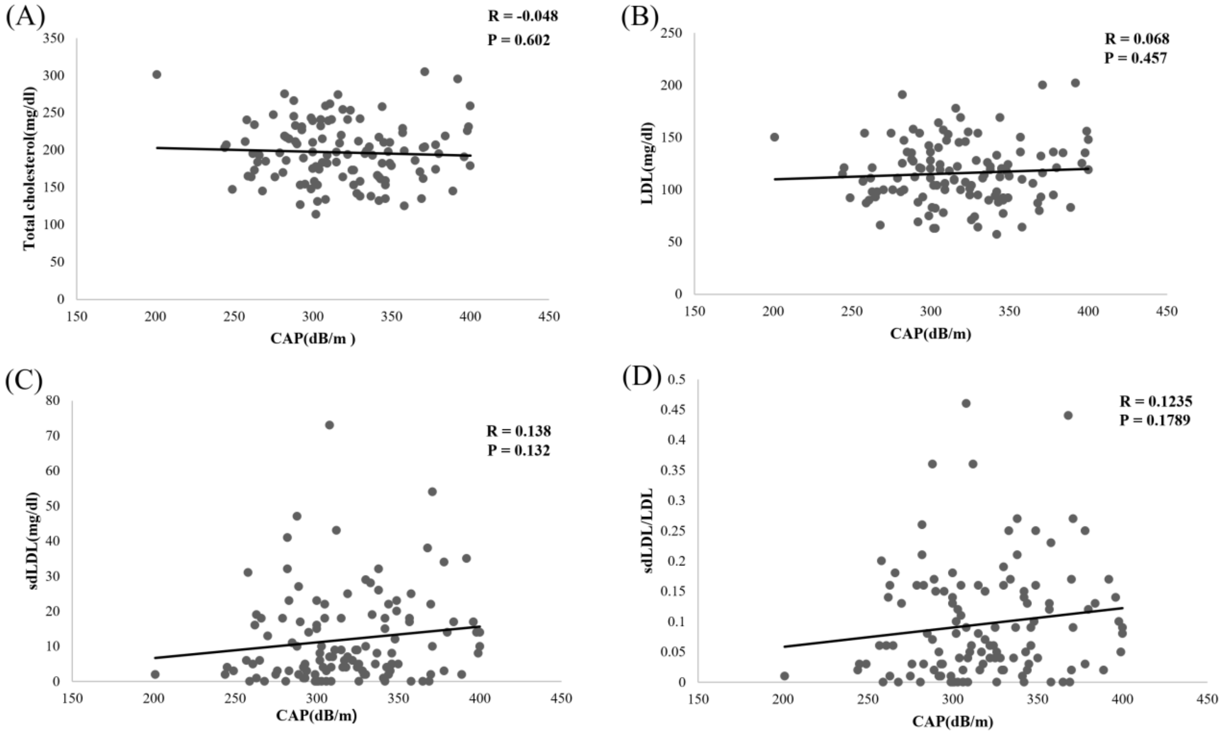

Supplement: Supplementary file 2 [file medi-101-e30527-s002.pdf]
